# Drug-induced kidney injury in Chinese critically ill pediatric patients

**DOI:** 10.3389/fphar.2022.993923

**Published:** 2022-09-26

**Authors:** Biwen Hu, Ling Ye, Tong Li, Zeying Feng, Longjian Huang, Chengjun Guo, Li He, Wei Tan, Guoping Yang, Zhiling Li, Chengxian Guo

**Affiliations:** ^1^ Center of Clinical Pharmacology, The Third Xiangya Hospital, Central South University, Changsha, Hunan, China; ^2^ West Guangxi Key Laboratory for Prevention and Treatment of High-Incidence Diseases, Youjiang Medical University for Nationalities, Baise, China; ^3^ School of Applied Mathematics, Guangdong University of Technology, Guangzhou, China; ^4^ Department of Pediatrics, The Third Xiangya Hospital, Central South University, Changsha, Hunan, China; ^5^ Department of Neonatology, Maternal and Child Health Hospital of Guangxi Zhuang Autonomous Region, Nanning, Guangxi Zhuang Region, China; ^6^ Department of Pharmacy, Shanghai Children’s Hospital, School of Medicine, Shanghai Jiao Tong University, Shanghai, China

**Keywords:** drug-induced kidney injury, acute kidney injury, rational drug use, rational medication, pediatrics

## Abstract

**Background:** Drug-induced acute kidney injury (DIKI) is a common adverse drug reaction event but is less known in pediatric patients. The study explored the DIKI in Chinese pediatric patients using the Pediatric Intensive Care database (PIC).

**Method:** We screened pediatric patients with acute kidney injury (AKI) using the KDIGO criteria from the PIC and then assessed the relationship between their drugs and DIKI using the Naranjo scale. For the fifteen frequently used DIKI-suspected drugs, we divided patients into drug-exposed and non-exposed groups, using the outcome of whether DIKI was presented or not. Propensity score matching (PSM) was used to control for the effects of four confounders, age, gender, length of hospital stay, and major diagnosis. Unconditional logistic regression was used to identify statistically significant differences between the two groups.

**Results:** A total of 238 drugs were used 1,863 times by the 81 patients with DIKI during their hospital stay. After screening the Naranjo scale to identify the top 15 suspected DIKI drugs with a high frequency of use, we found that furosemide injection (*p* = 0.001), midazolam injection (*p* = 0.001), 20% albumin prepared from human plasma injection (*p* = 0.004), fentanyl citrate injection (*p* = 0.001), compound glycyrrhizin injection (*p* = 0.026), vancomycin hydrochloride for intravenous (*p* = 0.010), and milrinone lactate injection (*p* = 0.009) were associated with DIKI.

**Conclusion:** In critically ill pediatric patients, DIKI is more likely to occur after using furosemide injection, midazolam injection, 20% albumin prepared from human plasma injection, fentanyl citrate injection, compound glycyrrhizin injection, vancomycin hydrochloride for intravenous, milrinone lactate injection.

## Introduction

AKI is a common problem in hospitalized children, and its overall incidence can be as high as 33.7% (Xu et al., 2018; Feiten et al., 2019). It is also associated with higher mortality and sequelae and may lead to chronic kidney disease in adulthood (Kaddourah et al., 2017; Levey and James, 2017). As the use of drugs is unavoidable in pediatric patients in intensive care, the identification and attention to nephrotoxic drugs play a key role in reducing morbidity as well as mortality in this population (Slater et al., 2017; Joyce et al., 2019). There has been a great deal of research into drug-induced kidney injury in adult inpatients. It is shown that the use of drugs such as NSAIDs and diuretics is an independent risk factor for AKI in adult hospitalized patients (Yu C. et al., 2020). The use of iodine-based contrast media can also lead to acute kidney injury during hospitalization and eventually to persistent renal insufficiency or end-stage renal disease (Cheng et al., 2020; Weisbord et al., 2020). In addition, prolonged antibiotic therapy with vancomycin also greatly increases the risk of acute kidney injury (O'Callaghan et al., 2020). Immune-related adverse events caused by immune checkpoint inhibitors when used in cancer treatment can affect the kidneys, leading to acute kidney injury associated with immune checkpoint inhibitor therapy (Meraz-Muñoz et al., 2020). There is also a series of studies on drug-induced kidney injury in pediatric inpatients. It was found that increased exposure to nephrotoxic drugs was associated with the development of AKI in pediatric inpatients (Searns et al., 2020). The use of drugs such as NSAIDs and proton pump inhibitors, which are more common in hospitalized children, also increases the risk of acute kidney injury during hospitalization (Xu et al., 2018; Su et al., 2021). Antibacterial drugs such as vancomycin and aminoglycoside antibiotics can affect renal function through different mechanisms of action, leading to acute kidney injury in children (Downes et al., 2017; Downes et al., 2020). Medication needs to be used with even greater caution in the complex conditions of the intensive care unit. However, there is still a lack of systematic research on the relationship between medications in this setting and the development of acute kidney injury.

Here, we used the PIC database to study 13,449 patients at the Children’s Hospital of Zhejiang University School of Medicine from 2010 to 2019 (Zeng et al., 2020), mainly collecting information on serum creatinine values and medication use of patients. The purpose of this study was to analyze the type and distribution of drugs for DIKI in pediatric patients and explain the occurrence of AKI by the mechanism of medications, to provide medical advice for children.

## Methods

### Study population

We conducted a retrospective case-control study, using the PIC database. The pediatric in-patients aged between 1 month and 18 years were included in the study (Ingelfinger et al., 2016; Xu et al., 2018). Patients with one of the following conditions were excluded: 1) age less than 1 month; 2) lacking records of prescriptions; 3) less than two serum creatinine tests; 4) baseline creatinine beyond the normal range; 5) an admitting diagnosis of acute renal failure (community-acquired AKI), chronic kidney disease, end-stage renal disease, renal transplantation, urinary tract infection, or pyelonephritis; 6) unexplained high serum creatinine values or low serum creatinine values (may be data entry errors). Physician-diagnosed AKI on admission or at discharge was determined according to the International Coding Definitions Version 10 (ICD-10).

### Data collection

Demographic data were abstracted from the database, including age and sex from ADMISSION, the name, and dose of drugs from PRESCRIPTION, and serum creatinine values from LABEVENT. When the difference between the two serum creatinine values met one of the Kidney Disease Improving Global Outcomes (KDIGO, 2012), patients were judged to have AKI. We considered that the AKI occurred when the earliest test of serum creatinine defining AKI happened. As the frequency of serum creatinine testing varied between pediatric patients, we also calculated and recorded the time difference between their serum creatinine test at the time of AKI and the previous one. Drug usage was defined as all the drugs the patient used before AKI.

### Identification of AKI

We used the KDIGO criteria to screen the patients with AKI in the PIC database, where AKI is defined as an increase in serum creatinine by 0.3 mg/dl within 48 h or a 50% increase in serum creatinine from the baseline within 7 days. Because no urine output was recorded in the database, we only used serum creatinine values to define AKI. The database only records patients’ data after admission, so we used the lowest serum creatinine level on the hospitalized days as the baseline serum creatinine level. Meanwhile, the baseline value was required to be within the normal range (Siew and Matheny, 2015).

### Relevance evaluation between drugs and AKI

Combined with patients’ drug usage during hospitalization, the Naranjo scale was used to further judge DIKI patients and DIKI suspected drugs on the AKI patients screened from the PIC database (Naranjo et al., 1981). The relevance evaluation of the criteria was: affirmative: ≥9 points; very likely: 5-8 points; possible: 1–4 points; impossible: ≤0 points. All cases with a score greater than 1 were considered DIKI, and the drugs which had a score greater than 1were considered DIKI-suspected signals.

### Validation of suspected drugs

After scoring the drugs using the Naranjo scale, we screened for suspected drugs associated with the occurrence of DIKI. Among these DIKI-suspected signals, the 15 most frequently used drugs were further analyzed. We divide patients from the PIC database into drug-exposed and non-exposed groups, with DIKI as an outcome. The inclusion and exclusion criteria for patients in both groups were consistent with the Identification of AKI section above. PSM was using for 15 drugs to adjust four confounders, including age, sex, length of stay, and major diagnosis, with two groups matched with a ratio of 1:1 by setting the caliper value (Yu Y. et al., 2020). After matching, unconditional logistic regression was performed on both groups to investigate the relationship between the suspected signals and DIKI. Chi-square test was used to compare mortality between the two groups.

### Statistical analysis

Descriptive analysis was used to present the medication information of the patients. Categorical variables are expressed using frequency. PSM was performed using R4.1.1 software, the “MatchIt” package version 4.4.0. The unconditional logistic regression and chi-square test were performed for the exposed and unexposed groups using SPSS version 18.0 statistical software. *p* < 0.05 indicates that the difference is statistically significant.

## Results

### Clinical characteristics of pediatric patients with DIKI

We screened 13,449 patients from the PIC database. A total of 178 patients met the age requirement and were rated as having developed AKI according to the KDIGO criteria. However, 79 patients with AKI were excluded from the study due to the absence of prescriptions. According to the Naranjo scale, 1 patient had a case score of 5 and was considered likely to have DIKI, and 80 patients had a case score of 1–4 and were considered likely to have DIKI, leaving 4 patients with a case score ≤0. After a careful review of the prescriptions for 85 patients with AKI, 81 patients considered to be potentially associated with DIKI were included in the study.

The clinical features of patients with DIKI are shown below (Table 1). 66.7% of the 81 patients included in the study with DIKI were male and 33.3% were female, giving a male to female ratio of 2:1. Overall, 45.7% of pediatric patients were aged 1 month to 1 year, 43.2% were aged 2–10 years, and 11.1% were aged 11–18 years. The top two major diseases were tumors (24.7%) and respiratory diseases (18.5%). Because of different status of the pediatric patients or incomplete database records, the frequency of serum creatinine testing was 42% (n = 34) of DIKI patients tested twice within 24h, 24.7% (n = 20) of patients tested once within 24h and 33.3% (n = 27) of patients tested once more than 48 h. There were 43.2% of DIKI patients in Stage 1, 24.7% in Stage 2, and 32.1% in Stage 3 according to the KDIGO criteria. Their median length of stay was 13 days (6, 22.5), and 48.1% of patients died during their stay. Before discharge or death, renal function was fully recovered in 17.3% of patients, partially recovered in 21.0% of patients, and not recovered in 61.7% of patients.

**TABLE 1:** Demographic information and clinical characteristics of patients with drug-induced kidney injury.

	N (%)
Gender	
Male	54 (66.7%)
Female	27 (33.3%)
Age	
Infancy,1 mo to 1 yr	37 (45.7%)
Childhood, 2–10 years	35 (43.2%)
Adolescence, 11–18 years	9 (11.1%)
Major Diagnosis	
Neoplasms	20 (24.7%)
Respiratory system	15 (18.5%)
Blood and blood-forming organs	8 (9.9%)
Congenital malformations, deformations and chromosomal abnormalities	8 (9.9%)
Circulatory system	7 (8.6%)
Nervous system	6 (7.4%)
Digestive system	5 (6.2%)
Injury, poisoning and certain other consequences of external causes	4 (4.9%)
Endocrine, nutritional and metabolic diseases	2 (2.5%)
musculoskeletal system and connective tissue	2 (2.5%)
Symptoms, signs and abnormal clinical and laboratory findings, not elsewhere classified	2 (2.5%)
Genitourinary system	1 (1.2%)
External causes of morbidity and mortality	1 (1.2%)
AKI stage	
Stage 1	35 (43.2%)
Stage 2	20 (24.7%)
Stage 3	26 (32.1%)
Length of stay, d	13 (6, 22.5)
In-hospital death	
Yes	39 (48.1%)
No	42 (51.9%)
Renal Recovery	
Full Recovery	14 (17.3%)
Partial Recovery	17 (21.0%)
Failure to Recovery	50 (61.7%)

Abbreviation: AKI, acute kidney injury.

For pediatric patients with different AKI Stage, their use of suspected DIKI drugs is largely similar (Supplementary Table S1). This is also shown in pediatric patients with different renal recovery (Supplementary Table S2).

### Screening for suspected DIKI signals

**FIGURE 1:**
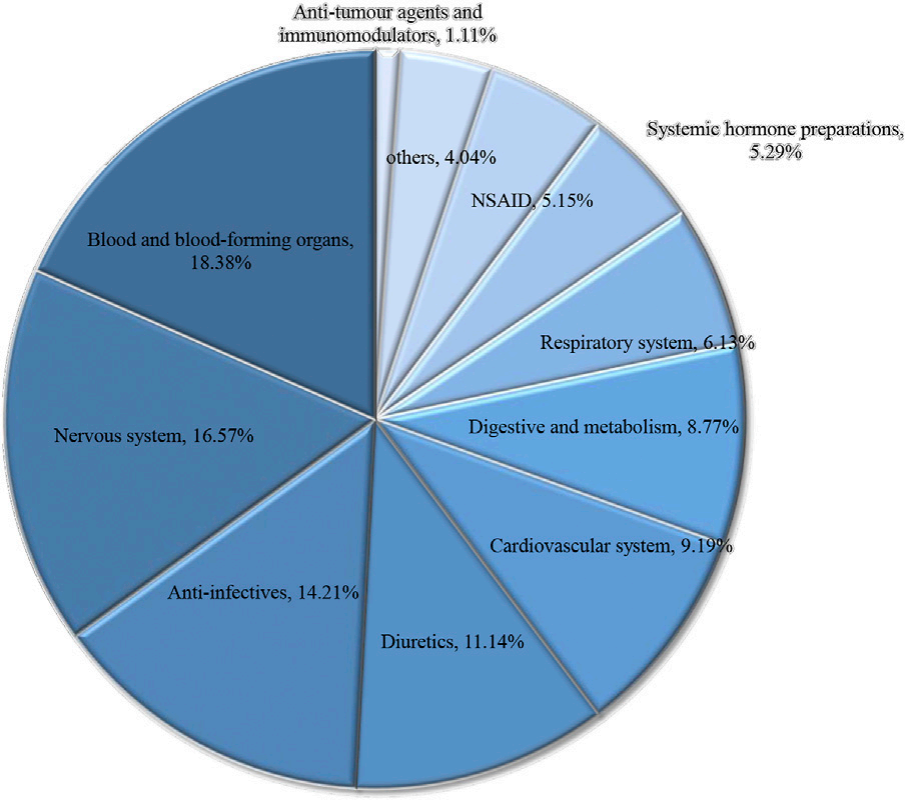
Drug categories and high frequency of drug for Pediatric patients with DIKI. Abbreviation: DIKI, drug-induced kidney injury.

A total of 238 drugs were used 1,863 times by 81 patients with DIKI during their hospital stay. We evaluated all drugs used before AKI using the Naranjo scale. Overall, The Naranjo score for 102 kinds of drugs was ≤0, for 134 kinds of was 1–4, for 2 kinds of drugs was 5. We considered that 136 kinds of drugs with Naranjo score greater than 1 were likely to be associated with the occurrence of AKI. About the 136 suspected DIKI drugs, 5.29% were systemic hormone preparations, 14.21% were anti-infectives, 5.15% were NSAIDs, 6.13% were respiratory system drugs, 1.11% were anti-tumor agents and immunomodulators, 16.57% were nervous system drugs, 8.77% were digestive and metabolism drugs, 11.14% were diuretics, 9.19% were cardiovascular system drugs, 18.38% were drugs related with the blood and blood-forming organs, and 4.04% belonged to other categories (Figure 1). After excluding drugs with Naranjo scores <0, the frequency of medication use in the 81 patients with DIKI was compiled (Supplementary Table S3).

### Validation of DIKI signals

**FIGURE 2:**
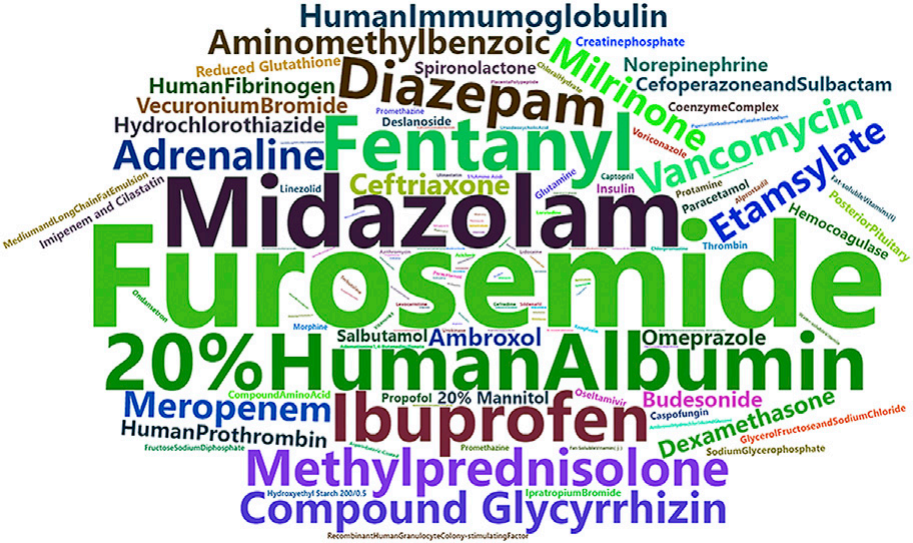
The suspected DIKI drugs. Abbreviation: DIKI, drug-induced kidney injury.

These drugs were used in the top 15 of all drugs used in the 81 patients with DIKI, with furosemide injection being the most frequently used (Figure 2). There were 1,417 cases in which furosemide injection was used, along with 1,096 cases with midazolam injection, 1,273 cases with 20% albumin prepared from human plasma injection, 792 cases with fentanyl citrate injection, 1,134 cases with ibuprofen suspension, 1,704 cases with diazepam injection, 1,162 cases with methylprednisolone sodium succinate for injection, 738 cases with compound glycyrrhizin injection, 468 cases with vancomycin hydrochloride for intravenous, 1,222 cases with adrenaline hydrochloride injection, 506 cases with milrinone lactate injection, 803 cases with meropenem for injection, 688 cases with human immumoglobulin for intravenous injection, 804 cases with aminomethylbenzoic acid injection, and 1,015 cases with etamsylate injection (Supplementary Table S4). After conducting statistics on them separately using logistic regression, we found that 7 drugs were found to be associated with the development of DIKI. Vancomycin hydrochloride for intravenous (*p* = 0.010, *OR* = 14.40, *95%CI* = 1.89–109.96) as a highly effective diuretic and nephrotoxin furosemide injection (*p* = 0.001, *OR* = 12.71, *95%CI* = 3.00–53.75) were strongly associated with DIKI. The pediatric patients in ICU using them independently increased the odds of DIKI. In our result, it appeared the anaesthetics midazolam injection (*p* = 0.001, *OR* = 8.16, *95%CI* = 2.45–27.17), fentanyl citrate injection (*p* = 0.001, *OR* = 7.87, *95%CI* = 2.35–26.31) could also increase the risk of DIKI. In additions, milrinone lactate injection (*p* = 0.009, *OR* = 7.17, *95%CI* = 1.62–31.72), 20% albumin prepared from human plasma injection (*p* = 0.004, *OR* = 4.25, *95%CI* = 1.60–11.32), and compound glycyrrhizin injection (*p* = 0.026, *OR* = 3.55, *95%CI* = 1.16–10.83) (Table 2). To explore the impact of drug use on patient mortality, we also compared the mortality rates of the two groups. We found that mortality rates were higher in the exposed group than in the unexposed group, except for milrinone lactate injection (Supplementary Table S5).

**TABLE 2:** Exposure and non-exposure of suspected drugs and the risk of DIKI.

Drug ID	Drugs name	Exposed group		Unexposed group		Beta	P	OR (95%CI)
		+	−	+	−			
1	Furosemide Injection	25	1,392	2	1,415	2.54	0.001	12.71 (3.00–53.75)
2	Midazolam Injection	24	1,072	3	1,093	2.10	0.001	8.16 (2.45–27.17)
3	20% Albumin Prepared From Human Plasma Injection	21	1,252	5	1,268	1.45	0.004	4.25 (1.60–11.32)
4	Fentanyl citrate Injection	23	769	3	789	2.06	0.001	7.87 (2.35–26.31)
5	Ibuprofen Suspension	14	1,120	7	1,127	0.70	0.132	2.01 (0.81–5.00)
6	Diazepam Injection	15	1,689	14	1,690	0.07	0.852	1.07 (0.52–2.23)
7	Methylprednisolone sodium succinate for Injection	13	1,149	9	1,153	0.37	0.394	1.45 (0.62–3.40)
8	Compound Glycyrrhizin Injection	14	724	4	734	1.27	0.026	3.55 (1.16–10.83)
9	Vancomycin Hydrochloride for Intra Venous	14	454	1	467	2.67	0.010	14.40 (1.89–109.96)
10	Adrenaline Hydrochlaride Injection	9	1,213	9	1,213	0.00	1.000	1.000 (0.40–2.53)
11	Milrinone Lactate Injection	14	492	2	504	1.97	0.009	7.17 (1.62–31.72)
12	Meropenem for Injection	10	793	9	794	0.11	0.818	1.11 (0.45–2.75)
13	Human Immumoglobulin for Intravenous Injection	11	677	7	681	0.46	0.347	1.52 (0.61–4.10)
14	Aminomethylbenzoic Acid Injection	10	794	9	795	0.11	0.818	1.11 (0.45–2.75)
15	Etamsylate Injection	9	1,006	11	1,004	−0.203	0.654	0.82 (0.34–1.98)

Abbreviation: DIKI, drug-induced kidney injury; OR, odds ratio.

## Discussion

In the current study, we evaluated patients in the PIC database for AKI according to the KDIGO criteria. Then, using the Naranjo scale, we determined the correlation between patients’ AKI and the medications used during hospitalization, and further validated the above judgments using statistical analysis. Most of the drugs that led to DIKI in patients belonged to drugs related to the blood and blood-forming organs, nervous system drugs, and diuretics (Figure 1). Meanwhile, among the suspected drugs associated with AKI, furosemide injection, midazolam injection, and 20% human albumin injections were considered to be highly associated with the occurrence of DIKI. This result deepens our understanding of nephrotoxic drugs and provides inspiration to search for new signals of DIKI.

After further validation of 15 drugs, we found that 7 drugs were considered to be correlated with the occurrence of AKI. They were furosemide injection, midazolam injection, 20% albumin prepared from human plasma injection, fentanyl citrate injection, compound glycyrrhizin injection, vancomycin hydrochloride for intravenous, and milrinone lactate injection. As two drugs closely associated with AKI, vancomycin hydrochloride for intravenous and furosemide injection have been the subject of many relevant studies so far. Numerous studies have shown that critically ill patients who have used vancomycin are more likely to develop AKI and that vancomycin-associated nephrotoxicity is often associated with higher serum vancomycin levels (Hanrahan et al., 2015; Feiten et al., 2019). The mechanism by which vancomycin causes the onset of AKI remains poorly understood, but it is currently believed that the oxidative effects of vancomycin lead to renal tubular ischemia (Elyasi et al., 2012). Pathology reports showing acute tubular necrosis have also been reported in pediatric patients with vancomycin-induced AKI who underwent renal biopsy (Wu et al., 2007). Unlike vancomycin, furosemide injection is not a nephrotoxic drug. It can be used to treat oedema caused by diseases such as congestive heart failure and cirrhosis (Joannidis et al., 2019; Zhao et al., 2020). When renal perfusion is inadequate, furosemide can reduce the potential risk of tubular injury due to ischaemia by inhibiting the Nn-2Cr co-transporter (NKCC2), reducing ion channel activity and decreasing cellular oxygen consumption. However, prior fluid replacement is required to correct hypovolemia, otherwise the use of furosemide in hypovolemic patients is likely to promote the development of AKI (Shah et al., 2017; Joannidis et al., 2019). It has also been demonstrated that the use of furosemide in pediatric patients in intensive care units increases the risk of AKI in patients (Slater et al., 2017). In our results, the proportion of patients who used furosemide injection and developed a DIKI with a preliminary diagnosis of heart disease was particularly low. Thus, we ruled out this possibility of AKI occurring as a result of cardio-renal syndrome. From our results, it appears that the use of furosemide can increase the risk of AKI in pediatric patients. Before using furosemide in critically ill patients, it is important to note the status of patients. The 20% albumin prepared from human plasma injection can be used to treat shock due to blood loss or burns, elevated cranial pressure due to cerebral edema and injury, or edema or ascites due to cirrhosis and renal disease. We can draw inspiration from the hypothesis of renal failure described by Moran and Kapsner (Moran and Kapsner, 1987): Glomerular Filtration Rate (GFR) is related to the imbalance of renal perfusion pressure and oncotic forces at the membrane of the glomeruli; when the colloid osmotic pressure increases, glomerular filtration decreases or stops. It can be used to correct blood volume deficiencies and maintain plasma colloid osmotic pressure. A studies found that the use of hypertonic albumin injections may increase the risk of AKI in patients needing fluid resuscitation for shock (Schortgen et al., 2008). In addition, a study had also found that albumin administration is associated with an increased risk of AKI after cardiac surgery in a dose-dependent manner (Frenette et al., 2014). However, as can be seen from the distribution of our patients’ major diagnosis (Table 1), the proportion of DIKI patients with heart disease was not high (8.6%). We also used PSM in the exposed and non-exposed groups to avoid the effect of the primary diagnosis on the outcome. When using 20% Human Albumin Injection, excessive blood volume may result once the infusion dose and infusion rate are not adjusted to the patient’s circulatory status. Continued supplementation of human albumin in the presence of excess blood volume can exacerbate the patient’s risk of AKI. Therefore, when using this drug, it is important to focus on the patient’s circulatory status first to prevent the development of DIKI. Midazolam is a sedative drug, which is commonly used for endoscopic procedures, general anesthesia, and sedation of patients with tracheal intubation and mechanical ventilation. Fentanyl citrate injection, a drug commonly used in compound general anesthesia today, is frequently used for sedation and analgesia before, during, and after anesthesia. Midazolam and fentanyl are commonly used in pediatric intensive care units (PICUs). The UK Pediatric Intensive Care Society’s consensus have recommended the combination of midazolam and fentanyl in continuous infusion as the first choice for sedation/ analgesia in PICUs patients (Playfor et al., 2006). Fentanyl belongs to the synthetic class of opioid receptor agonists. Opioids can cause kidney damage by causing excessive sedation, hypoxia and decreased renal blood flow in pediatric patients (Schenk et al., 1995; Feng et al., 2015; Mallappallil et al., 2017). Midazolam Injection belongs to the benzodiazepine group of drugs and is also often used in combination with fentanyl injection in our results, which can make it more dangerous and more likely to lead to excessive sedation and cause AKI. Therefore, pediatric patients should be alert to the development of AKI when using midazolam injection and fentanyl citrate injection, especially when they are used in combination. Compound glycyrrhizin injection can be used to treat chronic liver disease, improve abnormal liver function, and treat eczema, dermatitis, and urticaria. Its main component is glycyrrhizin, which is extracted from *Glycyrrhiza* glabra (Deutch et al., 2019). There are no studies on the association of Compound glycyrrhizin injection with the development of AKI and it is not clear what causes it to occur. However, its structure is similar to that of aldosterone, which enables it to mimic the sodium-retaining and potassium-removing effects of aldosterone. It can also inhibit the activity of 11β-hydroxysteroid dehydrogenase, leading to the increased release of other aldosterone-like components and resulting in the development of hypokalemia. Once hypokalaemia occurs, it may lead to dysfunction of the sodium-potassium pump, extracellular calcium entry of extracellular calcium into cells, increased intracellular free calcium ion concentration, activation of intracellular proteases by calcium overload, causing necrosis of myocytes and muscle fibres, release of harmful components into the extracellular fluid and blood, and ultimately renal impairment. Meanwhile, milrinone lactate injection is primarily used for the short-term treatment of patients with acute decompensated heart failure. In pediatric patients, it can be used to prevent cardiac output syndrome after cardiac surgery. These patients are likely to be treated with Cardiopulmonary bypass (CPB) at the same time. It has been reported that there is a significant association between the perioperative use of milrinone and acute kidney risk or injury in pediatric patients who have received CPB (Chiravuri et al., 2011). However, CPB has also been shown to be a risk factor for the development of AKI in pediatric patients (Aydin et al., 2012; Pedersen, 2012). Thus, we cannot clarify the effect of milrinone lactate injection on the development of AKI in pediatric patients other than the effect of CPB, but the use of milrinone injection in pediatric patients receiving CPB may also increase the risk of AKI.

In our study, there are several shortcomings. First of all, due to the small sample of patients, it is hard to explore the factors influencing adverse drug reactions. It is an urgent need for larger population-based studies to validate and further investigate the drugs that we found to be associated with the occurrence of AKI. Secondly, as it was a retrospective study using a secondary database, the information recorded in the database on the patients will not exactly match the purpose of our study, and for these patients we need to exclude. Half of patients were excluded due to missing records of medication information or apparently incorrect records of data. Third, there are many factors that can contribute to the development of AKI in the PICU and medication is only one part of them. We learned that some factors such as hemodynamic status and cardiac surgery were risk factors for the development of AKI, but we were unable to include these important factors in our analysis because no such information was available in the database for the duration of the patient’s hospitalization.

Our study found that furosemide injection, midazolam injection, 20% albumin prepared from human plasma injection, fentanyl citrate injection, compound glycyrrhizin injection, vancomycin hydrochloride for intravenous and milrinone lactate injection were risk factors for the development of AKI in critically ill pediatric patients. These results suggest that, because of the many complex factors that may be encountered in critical care units, the administration of medications for these patients needs to be more rigorous and rational, according to their own conditions, to avoid adverse effects caused by these drugs, as much as possible.

## Conclusion

In order to search for drugs associated with critically ill pediatric patients with DIKI, we assessed and validated the relationship between patients and suspected drugs, screening patients in the PIC database, finally we found some drugs related to DIKI. We hope to provide some reference for physicians and pharmacists when administering drugs to critically ill pediatric patients to reduce adverse reactions of drugs.

## Data Availability

The raw data supporting the conclusions of this article will be made available by the authors, without undue reservation.

## References

[B1] Aydin S. I. Seiden H. S. Blaufox A. D. Parnell V. A. Choudhury T. Punnoose A. (2012). Acute kidney injury after surgery for congenital heart disease. Ann. Thorac. Surg. 94 (5), 1589–1595. 10.1016/j.athoracsur.2012.06.050 22884599

[B2] Cheng W. Wu X. Liu Q. Wang H. S. Zhang N. Y. Xiao Y. Q. (2020). Post-contrast acute kidney injury in a hospitalized population: Short-mid-and long-term outcome and risk factors for adverse events. Eur. Radiol. 30 (6), 3516–3527. 10.1007/s00330-020-06690-3 32080754 PMC7248019

[B3] Chiravuri S. D. Riegger L. Q. Christensen R. Butler R. R. Malviya S. Tait A. R. (2011). Factors associated with acute kidney injury or failure in children undergoing cardiopulmonary bypass: A case-controlled study. Paediatr. Anaesth. 21 (8), 880–886. 10.1111/j.1460-9592.2011.03532.x 21306475

[B4] Deutch M. R. Grimm D. Wehland M. Infanger M. Krüger M. (2019). Bioactive candy: Effects of licorice on the cardiovascular system. Foods 8 (10), E495. 10.3390/foods8100495 31615045 PMC6836258

[B5] Downes K. J. Cowden C. Laskin B. L. Huang Y. S. Gong W. Bryan M. (2017). Association of acute kidney injury with concomitant vancomycin and piperacillin/tazobactam treatment among hospitalized children. JAMA Pediatr. 171 (12), e173219. 10.1001/jamapediatrics.2017.3219 28973124 PMC6583633

[B6] Downes K. J. Hayes M. Fitzgerald J. C. Pais G. M. Liu J. Zane N. R. (2020). Mechanisms of antimicrobial-induced nephrotoxicity in children. J. Antimicrob. Chemother. 75 (1), 1–13. 10.1093/jac/dkz325 31369087 PMC6910165

[B7] Elyasi S. Khalili H. Dashti-Khavidaki S. Mohammadpour A. (2012). Vancomycin-induced nephrotoxicity: Mechanism, incidence, risk factors and special populations. A literature review. Eur. J. Clin. Pharmacol. 68 (9), 1243–1255. 10.1007/s00228-012-1259-9 22411630

[B8] Feiten H. D. S. Okumura L. M. Martinbiancho J. K. Andreolio C. Da Rocha T. S. Antonacci Carvalho P. R. (2019). Vancomycin-associated nephrotoxicity and risk factors in critically ill children without preexisting renal injury. Pediatr. Infect. Dis. J. 38 (9), 934–938. 10.1097/inf.0000000000002391 31232892

[B9] Feng G. Luo Q. Guo E. Yao Y. Yang F. Zhang B. (2015). Multiple organ dysfunction syndrome, an unusual complication of heroin intoxication: A case report and review of literature. Int. J. Clin. Exp. Pathol. 8 (9), 11826–11830. 26617935 PMC4637751

[B10] Frenette A. J. Bouchard J. Bernier P. Charbonneau A. Nguyen L. T. Rioux J. P. (2014). Albumin administration is associated with acute kidney injury in cardiac surgery: A propensity score analysis. Crit. Care 18 (6), 602. 10.1186/s13054-014-0602-1 25394836 PMC4256900

[B11] Hanrahan T. P. Kotapati C. Roberts M. J. Rowland J. Lipman J. Roberts J. A. (2015). Factors associated with vancomycin nephrotoxicity in the critically ill. Anaesth. Intensive Care 43 (5), 594–599. 10.1177/0310057x1504300507 26310409

[B12] Ingelfinger J. R. Kalantar-Zadeh K. Schaefer F. (2016). Ingelfinger JR, kalantar-zadeh K, schaefer F; for the world kidney day steering committee. Averting the legacy of kidney disease-focus on childhood. Kidney int. 2016;89:512-518. Kidney Int. 89 (6), 1405. 10.1016/j.kint.2016.04.001 27181786

[B13] Joannidis M. Klein S. J. Ostermann M. (2019). 10 myths about frusemide. Intensive Care Med. 45 (4), 545–548. 10.1007/s00134-018-5502-4 30643933

[B14] Joyce E. L. Kane-Gill S. L. Priyanka P. Fuhrman D. Y. Kellum J. A. (2019). Piperacillin/Tazobactam and antibiotic-associated acute kidney injury in critically ill children. J. Am. Soc. Nephrol. 30 (11), 2243–2251. 10.1681/asn.2018121223 31501354 PMC6830781

[B15] Kaddourah A. Basu R. K. Bagshaw S. M. Goldstein S. L. (2017). Epidemiology of acute kidney injury in critically ill children and young adults. N. Engl. J. Med. 376 (1), 11–20. 10.1056/NEJMoa1611391 27959707 PMC5322803

[B16] KDIGO (2012). Kidney disease: Improving global outcomes (KDIGO) acute kidney injury work group. KDIGO clinical practice guideline for acute kidney injury. Kidney Int. Suppl. 2 (1), 1–138. 10.1038/kisup.2012.1

[B17] Levey A. S. James M. T. (2017). Acute kidney injury. Ann. Intern. Med. 167 (9), ITC66-ITC80–itc80. 10.7326/aitc201711070 29114754

[B18] Mallappallil M. Sabu J. Friedman E. A. Salifu M. (2017). What do we know about opioids and the kidney? Int. J. Mol. Sci. 18 (1), E223. 10.3390/ijms18010223 28117754 PMC5297852

[B19] Meraz-Muñoz A. Amir E. Ng P. Avila-Casado C. Ragobar C. Chan C. (2020). Acute kidney injury associated with immune checkpoint inhibitor therapy: Incidence, risk factors and outcomes. J. Immunother. Cancer 8 (1), e000467. 10.1136/jitc-2019-000467 32601079 PMC7326260

[B20] Moran M. Kapsner C. (1987). Acute renal failure associated with elevated plasma oncotic pressure. N. Engl. J. Med. 317 (3), 150–153. 10.1056/nejm198707163170306 2439908

[B21] Naranjo C. A. Busto U. Sellers E. M. Sandor P. Ruiz I. Roberts E. A. (1981). A method for estimating the probability of adverse drug reactions. Clin. Pharmacol. Ther. 30 (2), 239–245. 10.1038/clpt.1981.154 7249508

[B22] O'callaghan K. Hay K. Lavana J. Mcnamara J. F. (2020). Acute kidney injury with combination vancomycin and piperacillin-tazobactam therapy in the ICU: A retrospective cohort study. Int. J. Antimicrob. Agents 56 (1), 106010. 10.1016/j.ijantimicag.2020.106010 32413387

[B23] Pedersen K. (2012). Acute kidney injury in children undergoing surgery for congenital heart disease. Eur. J. Pediatr. Surg. 22 (6), 426–433. 10.1055/s-0032-1322540 22903254

[B24] Playfor S. Jenkins I. Boyles C. Choonara I. Davies G. Haywood T. (2006). Consensus guidelines on sedation and analgesia in critically ill children. Intensive Care Med. 32 (8), 1125–1136. 10.1007/s00134-006-0190-x 16699772

[B25] Schenk H. D. Radke J. Ensink F. B. Drobnik L. Kettler D. Sonntag H. (1995). Interactions between renal and general hemodynamics in fentanyl, droperidol, ketamine, thiopental and in peridural anesthesia--animal studies. Anaesthesiol. Reanim. 20 (3), 60–70. 8526961

[B26] Schortgen F. Girou E. Deye N. Brochard L. (2008). The risk associated with hyperoncotic colloids in patients with shock. Intensive Care Med. 34 (12), 2157–2168. 10.1007/s00134-008-1225-2 18685828

[B27] Searns J. B. Gist K. M. Brinton J. T. Pickett K. Todd J. Birkholz M. (2020). Impact of acute kidney injury and nephrotoxic exposure on hospital length of stay. Pediatr. Nephrol. 35 (5), 799–806. 10.1007/s00467-019-04431-3 31940070

[B28] Shah R. Wood S. J. Khan S. A. Chaudhry A. Rehan Khan M. Morsy M. S. (2017). High-volume forced diuresis with matched hydration using the RenalGuard system to prevent contrast-induced nephropathy: A meta-analysis of randomized trials. Clin. Cardiol. 40 (12), 1242–1246. 10.1002/clc.22817 29247527 PMC6490568

[B29] Siew E. D. Matheny M. E. (2015). Choice of reference serum creatinine in defining acute kidney injury. Nephron 131 (2), 107–112. 10.1159/000439144 26332325 PMC4618709

[B30] Slater M. B. Gruneir A. Rochon P. A. Howard A. W. Koren G. Parshuram C. S. (2017). Identifying high-risk medications associated with acute kidney injury in critically ill patients: A pharmacoepidemiologic evaluation. Paediatr. Drugs 19 (1), 59–67. 10.1007/s40272-016-0205-1 27943125

[B31] Su L. Li Y. Xu R. Luo F. Gao Q. Chen R. (2021). Association of ibuprofen prescription with acute kidney injury among hospitalized children in China. JAMA Netw. Open 4 (3), e210775. 10.1001/jamanetworkopen.2021.0775 33662136 PMC7933997

[B32] Weisbord S. D. Palevsky P. M. Kaufman J. S. Wu H. Androsenko M. Ferguson R. E. (2020). Contrast-associated acute kidney injury and serious adverse outcomes following angiography. J. Am. Coll. Cardiol. 75 (11), 1311–1320. 10.1016/j.jacc.2020.01.023 32192658

[B33] Wu C. Y. Wang J. S. Chiou Y. H. Chen C. Y. Su Y. T. (2007). Biopsy proven acute tubular necrosis associated with vancomycin in a child: Case report and literature review. Ren. Fail. 29 (8), 1059–1061. 10.1080/08860220701643773 18067058

[B34] Xu X. Nie S. Zhang A. Mao J. Liu H. P. Xia H. (2018). Acute kidney injury among hospitalized children in China. Clin. J. Am. Soc. Nephrol. 13 (12), 1791–1800. 10.2215/cjn.00800118 30287424 PMC6302328

[B35] Yu C. Guo D. Yao C. Yang H. Liu S. Zhu Y. (2020a). Clinical characteristics of hospitalized patients with drug-induced acute kidney injury and associated risk factors: A case-control study. Biomed. Res. Int. 2020, 9742754. 10.1155/2020/9742754 33015190 PMC7512068

[B36] Yu Y. Nie X. Song Z. Xie Y. Zhang X. Du Z. (2020b). Signal detection of potentially drug-induced liver injury in children using electronic health records. Front. Pediatr. 8, 171. 10.3389/fped.2020.00171 32373564 PMC7177017

[B37] Zeng X. Yu G. Lu Y. Tan L. Wu X. Shi S. (2020). PIC, a paediatric-specific intensive care database. Sci. Data 7 (1), 14. 10.1038/s41597-020-0355-4 31932583 PMC6957490

[B38] Zhao G. J. Xu C. Ying J. C. Lü W. B. Hong G. L. Li M. F. (2020). Association between furosemide administration and outcomes in critically ill patients with acute kidney injury. Crit. Care 24 (1), 75. 10.1186/s13054-020-2798-6 32131879 PMC7057586

