# Supplementary material for: Drug-induced kidney injury in Chinese critically ill pediatric patients
Source: Front Pharmacol. 2022 Sep 26;13:993923. doi: 10.3389/fphar.2022.993923 (PMC9548562; doi:10.3389/fphar.2022.993923)
Supplement: Supplementary file 1 [file DataSheet1.docx]

**Table S1 Top 10 drugs used by DIKI patients with different AKI Stage**

| AKI Stage 1 (n=35) | | AKI Stage 2 (n=20) | | AKI Stage 3 (n=26) | |
| --- | --- | --- | --- | --- | --- |
| Drug | Frequency | Drug | Frequency | Drug | Frequency |
| Furosemide Injection | 60.00% | Furosemide Injection | 90.00% | Furosemide Injection | 76.92% |
| 20% Albumin Prepared From Human Plasma Injection | 42.86% | 20% Albumin Prepared From Human Plasma Injection | 40.00% | Midazolam Injection | 69.23% |
| Midazolam Injection | 40.00% | Fentanyl Citrate Injection | 40.00% | Fentanyl Citrate Injection | 46.15% |
| Ibuprofen Suspension | 34.29% | Midazolam Injection | 40.00% | Methylprednisolone Sodium Succinate for Injection | 38.46% |
| Diazepam Injection | 34.29% | Ibuprofen Suspension | 35.00% | 20% Albumin Prepared From Human Plasma Injection | 30.77% |
| Fentanyl Citrate Injection | 34.29% | Dexamethasone Sodium Phosphate Injection | 30.00% | Ibuprofen Suspension | 30.77% |
| Compound Glycyrrhizin Injection | 25.71% | Human Fibrinogen | 30.00% | Diazepam Injection | 26.92% |
| Meropenem for Injection | 25.71% | Human Prothrombin Complex | 30.00% | Vancomycin Hydrochloride for Intra Venous | 26.92% |
| Adrenaline Hydrochlaride Injection | 22.86% | Milrinone Lactate Injection | 30.00% | Compound Glycyrrhizin Injection | 23.08% |
| Etamsylate Injection | 20.00% | Diazepam Injection | 25.00% | Milrinone Lactate Injection | 23.08% |

Abbreviation: DIKI, drug-induced kidney injury; AKI, acute kidney injury

**Table S2 Top 10 drugs used by DIKI patients with different Renal Recovery**

| Full Recovery (n=14) | | Partial Recovery (n=17) | | Failure to Recovery (n=50) | |
| --- | --- | --- | --- | --- | --- |
| Drug | Frequency | Drug | Frequency | Drug | Frequency |
| Furosemide Injection | 71.43% | Furosemide Injection | 76.47% | Furosemide Injection | 74.00% |
| Fentanyl Citrate Injection | 42.86% | Midazolam Injection | 58.82% | Midazolam Injection | 52.00% |
| Midazolam Injection | 35.71% | Fentanyl Citrate Injection | 47.06% | 20% Albumin Prepared From Human Plasma Injection | 44.00% |
| 20% Albumin Prepared From Human Plasma Injection | 28.57% | Ibuprofen Suspension | 41.18% | Fentanyl Citrate Injection | 36.00% |
| Ibuprofen Suspension | 21.43% | 20% Albumin Prepared From Human Plasma Injection | 35.29% | Ibuprofen Suspension | 36.00% |
| Diazepam Injection | 21.43% | Diazepam Injection | 35.29% | Methylprednisolone Sodium Succinate for Injection | 36.00% |
| Budesonide Suspension for Inhalation | 21.43% | Meropenem for Injection | 29.41% | Diazepam Injection | 30.00% |
| Vecuronium Bromide for Injection | 14.29% | Vancomycin Hydrochloride for Intra Venous | 23.53% | Adrenaline Hydrochlaride Injection | 28.00% |
| Meropenem for Injection | 14.29% | Ceftriaxone Sodium for Injection | 17.65% | Compound Glycyrrhizin Injection | 26.00% |
| Human Immumoglobulin for Intravenous Injection | 14.29% | Compound Glycyrrhizin Injection | 17.65% | Milrinone Lactate Injection | 26.00% |

Abbreviation: DIKI, drug-induced kidney injury

**Table S3 Drug use in pediatric patients with DIKI (n=718)**

| Type of drug | Drug name | number of use (%) |
| --- | --- | --- |
| Anti-infectives | Vancomycin Hydrochloride for Intra Venous | 17 (2.37%) |
|  | Meropenem for Injection | 15 (2.09%) |
|  | Human Immumoglobulin for Intravenous Injection | 13 (1.81%) |
|  | Ceftriaxone Sodium for Injection | 12 (1.67%) |
|  | Cefoperazone Sodium and Sulbactam Sodium for Injection | 8 (1.11%) |
|  | Imipenem and Cilastatin Sodium for Injection | 5 (0.70%) |
|  | Oseltamivir Phosphate Capsules | 4 (0.56%) |
|  | Linezolid Injection | 4 (0.56%) |
|  | Voriconazole for Injection | 4 (0.56%) |
|  | Caspofungin Acetate for Injection | 4 (0.56%) |
|  | Aciclovir for Injection | 2 (0.28%) |
|  | Piperacillin Sodium and Tazobactam Sodium for Injection | 2 (0.28%) |
|  | Cefradine for Injection | 2 (0.28%) |
|  | Azithromycin for Injection | 2 (0.28%) |
|  | Aciclovir for Injection | 1 (0.14%) |
|  | Rifampicin Capsules | 1 (0.14%) |
|  | Ribavirin for Injection | 1 (0.14%) |
|  | Cefuroxime Sodium for Injection | 1 (0.14%) |
|  | Levofloxacin and Sodium Chloride Injection | 1 (0.14%) |
|  | Fluconazole Injection | 1 (0.14%) |
|  | Metronidazole Disodium Phosphate for Injection | 1 (0.14%) |
|  | Tigecycline for Injection | 1 (0.14%) |
| Anti-tumour agents and immunomodulators | Recombinant Human Granulocyte Colony-stimulating Factor Injection | 3 (0.42%) |
|  | Ciclosporin Soft Capsules | 1 (0.14%) |
|  | Ciclosporin Injection | 1 (0.14%) |
|  | Homoharringtonine Injection | 1 (0.14%) |
|  | Tacrolimus Capsules | 1 (0.14%) |
|  | Recombinant Human Granulocyte Colony-stimulating Factor Injection | 1 (0.14%) |
| Blood and blood-forming organs | 20% Albumin Prepared From Human Plasma Injection | 33 (4.60%) |
|  | Etamsylate Injection | 16 (2.23%) |
|  | Aminomethylbenzoic Acid Injection | 14 (1.95%) |
|  | Human Prothrombin Complex | 9 (1.25%) |
|  | Human Fibrinogen | 9 (1.25%) |
|  | 20% Mannitol Injection | 7 (0.97%) |
|  | Hemocoagulase For Injection | 6 (0.84%) |
|  | Sodium Glycerophosphate Injection | 4 (0.56%) |
|  | Pediatric Compound Amino Acid Injection(19AA-1) | 4 (0.56%) |
|  | Lyophilizing Thrombin Powder | 4 (0.56%) |
|  | Medium and Long Chain Fat Emulsion Injection | 4 (0.56%) |
|  | Glutamine for Injection | 4 (0.56%) |
|  | Hydroxyethyl Starch 200/0.5 and Sodium Chloride Injection | 3 (0.42%) |
|  | Aspirin Enteric-Coated Tablets | 2 (0.28%) |
|  | Lidocaine Hydrochloride Injection | 2 (0.28%) |
|  | 5% Amino Acidi Injection | 2 (0.28%) |
|  | Vitamin K1 Injection | 2 (0.28%) |
|  | Urokinase for Injection | 2 (0.28%) |
|  | Mecobalamin Tablets | 1 (0.14%) |
|  | ω-3 Fish Oil Fat Emulsion Injection | 1 (0.14%) |
|  | Arginine Hydrochloride Injection | 1 (0.14%) |
|  | Recombinant HumanThrombopoietin Injection | 1 (0.14%) |
|  | Haemocoagulase Agkistrodon for Injection | 1 (0.14%) |
| Cardiovascular system | Adrenaline Hydrochlaride Injection | 17 (2.37%) |
|  | Milrinone Lactate Injection | 16 (2.23%) |
|  | Norepinephrine Bitartrate Injection | 8 (1.11%) |
|  | Deslanoside Injection | 5 (0.70%) |
|  | Creatine phosphate sodium for injection | 5 (0.70%) |
|  | Captopril Tablets | 3 (0.42%) |
|  | Fructose Sodium Diphosphate Oral Solution | 3 (0.42%) |
|  | Alprostadil Injection | 3 (0.42%) |
|  | Digoxin Oral Solution | 1 (0.14%) |
|  | Nifedipine Tablets | 1 (0.14%) |
|  | Nitroglycerin Injection | 1 (0.14%) |
|  | Amiodarone Hydrochloride injection | 1 (0.14%) |
|  | Dopamine Hydrochloride Injection | 1 (0.14%) |
|  | Propafenone Hydrochloride Injection | 1 (0.14%) |
| Digestive and metabolism | Compound Glycyrrhizin Injection | 18 (2.51%) |
|  | Omeprazole Sodium For Injection | 9 (1.25%) |
|  | Reduced Glutathione Sodium for Injection | 6 (0.84%) |
|  | Insulin Injection | 5 (0.70%) |
|  | Ondansetron Hydrochloride Injection | 3 (0.42%) |
|  | Fat-soluble Vitamins for Injection(I) | 3 (0.42%) |
|  | Ademetionine 1,4-Butanedisulfonate for Injection | 2 (0.28%) |
|  | Ursodeoxycholic Acid Capsules | 2 (0.28%) |
|  | Fat-Soluble Vitamin Injection(Ⅱ) | 2 (0.28%) |
|  | Water-soluble Vitamin for Injection | 2 (0.28%) |
|  | Ulinastatin for Injection | 2 (0.28%) |
|  | Lactobacillus LB sachets | 1 (0.14%) |
|  | Montmorillonite Powder | 1 (0.14%) |
|  | Cimetidine Injection | 1 (0.14%) |
|  | Compound Glycyrrhizin Tablets | 1 (0.14%) |
|  | Lactulose Oral Solution | 1 (0.14%) |
|  | Vitamin B6 Injection | 1 (0.14%) |
|  | Vitamin C Injection | 1 (0.14%) |
|  | Metoclopramide Dihydrochloride Injection | 1 (0.14%) |
|  | Palonosetron hydrochloride Injection | 1 (0.14%) |
| Diuretics | Furosemide Injection | 60 (8.36%) |
|  | Hydrochlorothiazide Tablets | 9 (1.25%) |
|  | Spironolactone Tablets | 7 (0.97%) |
|  | Glycerol Fructose and Sodium Chloride Injection | 4 (0.56%) |
| Nervous system | Midazolam Injection | 41 (5.71%) |
|  | Fentanyl Citrate Injection | 32 (4.46%) |
|  | Diazepam Injection | 24 (3.34%) |
|  | Propofol Injectable Emulsion | 5 (0.70%) |
|  | Morphine Hydrachloride Injection | 3 (0.42%) |
|  | 10% Chloral Hydrate Enemas | 3 (0.42%) |
|  | Chlorpromazine Hydrochloride Injection | 2 (0.28%) |
|  | Oral Chloral Hydrate | 1 (0.14%) |
|  | Cattle Encephalon Glycoside and Ignotin Injection | 1 (0.14%) |
|  | 10% Chloral Hydrate Syrup | 1 (0.14%) |
|  | Sodium Valproate for Injection | 1 (0.14%) |
|  | Compound Lidocaine Cream | 1 (0.14%) |
|  | Pentazocine Injection | 1 (0.14%) |
|  | Pethidine Hydrochloride Injection | 1 (0.14%) |
|  | Dexmedetomidine Hydrochloride Injection | 1 (0.14%) |
|  | Phenobarbital Sodium for Injection | 1 (0.14%) |
| NSAID | Ibuprofen Suspension | 28 (3.90%) |
|  | Paracetamol Suspension Drop | 5 (0.70%) |
|  | Paracetamol Oral Solution | 2 (0.28%) |
|  | Paediatric Ibuprofen Suppositories | 1 (0.14%) |
|  | Lysine Acetylsalicylate for Injection | 1 (0.14%) |
| others | Vecuronium Bromide for Injection | 9 (1.25%) |
|  | Coenzyme Complex for Injection | 5 (0.70%) |
|  | Protamine Sulfate Injection | 4 (0.56%) |
|  | Levocarnitine Injection | 2 (0.28%) |
|  | Sildenafil Citrate Tablets | 2 (0.28%) |
|  | Placenta Polypeptide Injection | 2 (0.28%) |
|  | Kangfuxin Solution | 2 (0.28%) |
|  | Calcium Folinate for Injection | 1 (0.14%) |
|  | Relingqing Keli(sugar-free) | 1 (0.14%) |
|  | Wuwei Niuhuangsuan Koufuye | 1 (0.14%) |
| Respiratory system | Ambroxol Hydrochloride Injection | 10 (1.39%) |
|  | Budesonide Suspension for Inhalation | 9 (1.25%) |
|  | Salbutamol Sulfate Nebules Inhalation Solution | 7 (0.97%) |
|  | Ipratropium Bromide Solution for Inhalation | 4 (0.56%) |
|  | Promethazine Hydrochloride Tablets | 3 (0.42%) |
|  | Promethazine Hydrochloride Injection | 3 (0.42%) |
|  | Ambroxol Hydrochloride and Glucose Injection | 2 (0.28%) |
|  | Terbutaline Sulphate Solution for Nebulization | 2 (0.28%) |
|  | Loratadine Tablets | 2 (0.28%) |
|  | Aminophyiline Injection | 1 (0.14%) |
|  | Beclomethasone dipropionate inhalation suspension | 1 (0.14%) |
| Systemic hormone preparations | Methylprednisolone Sodium Succinate for Injection | 21 (2.92%) |
|  | Dexamethasone Sodium Phosphate Injection | 10 (1.39%) |
|  | Posterior Pituitary Injection | 5 (0.70%) |
|  | Hydrocortisone Sodium Succinate For Injection | 1 (0.14%) |
|  | Somatostatin For Injection | 1 (0.14%) |

Abbreviation: DIKI, drug-induced kidney injury

**Table S4 The clinical characteristic of patients in the exposed group and unexposed group**

| Factors |  | Furosemide Injection | | Midazolam Injection | | 20% Albumin Prepared From Human Plasma Injection | | Fentanyl Citrate Injection | | Ibuprofen Suspension | | Diazepam Injection | |
| --- | --- | --- | --- | --- | --- | --- | --- | --- | --- | --- | --- | --- | --- |
|  |  | EG | UEG | EG | UEG | EG | UEG | EG | UEG | EG | UEG | EG | UEG |
| n |  | 1417 | 1417 | 1096 | 1096 | 1273 | 1273 | 792 | 792 | 1134 | 1134 | 1704 | 1704 |
| caliper |  | 0.0007 | | 0.0010 | | 0.0010 | | 0.0012 | | 0.0010 | | 0.0013 | |
| Age | 1 month - 1 year | 809  (57.1) | 837  (59.1) | 685  (62.5) | 674  (61.5) | 714  (56.1) | 729  (57.3) | 518  (65.4) | 522  (65.9) | 612  (54.0) | 605  (53.4) | 1011  (59.3) | 1023  (60.0) |
|  | 1 year – 10 years | 515  (36.3) | 507  (35.8) | 337  (30.7) | 355  (32.4) | 484  (38.0) | 468  (36.8) | 228  (28.8) | 236  (29.8) | 451  (39.8) | 449  (39.6) | 546  (32.0) | 546  (32.0) |
|  | 11 years – 18 years | 93  (6.6) | 73  (5.2) | 74  (6.8) | 67  (6.1) | 75  (5.9) | 76  (6.0) | 46  (5.8) | 34  (4.3) | 71  (6.3) | 80  (7.1) | 147  (8.6) | 135  (7.9) |
|  | *P* | 0.229 | | 0.636 | | 0.549 | | 0.377 | | 0.748 | | 0.748 | |
| Gender (%) | Male | 826  (58.3) | 813  (57.4) | 617  (56.3) | 655  (59.8) | 711  (55.9) | 727  (57.1) | 463  (58.5) | 476  (60.1) | 626  (55.2) | 608  (53.6) | 972  (57.0) | 1006  (59.0) |
|  | Female |  |  |  |  |  |  |  |  |  |  |  |  |
|  | *P* | 0.648 | | 0.109 | | 0.549 | | 0.539 | | 0.474 | | 0.252 | |
| Major dignosis  (%) | Certain infectious and parastic diseases | 51  (3.6) | 37  (2.6) | 53  (4.8) | 49  (4.5) | 65  (5.1) | 54  (4.2) | 36  (4.5) | 35  (4.4) | 53  (4.7) | 50  (4.4) | 66  (3.9) | 69  (4.0) |
|  | Neoplasms | 128  (9.0) | 133  (9.4) | 82  (7.5) | 99  (9.0) | 111  (8.7) | 105  (8.2) | 56  (7.1) | 64  (8.1) | 106  (9.3) | 101  (8.9) | 111  (6.5) | 142  (8.3) |
|  | Diseases of the blood and blood-forming organs involving the immune mechanism | 45  (3.2) | 40  (2.8) | 24  (2.2) | 45  (4.1) | 40  (3.1) | 39  (3.1) | 25  (3.2) | 33  (4.2) | 42  (3.7) | 47  (4.1) | 52  (3.1) | 45  (2.6) |
|  | Endocrine,nutritional and metabolic diseases | 18  (1.3) | 20  (1.4) | 10  (0.9) | 16  (1.5) | 9  (0.7) | 7  (0.5) | 5  (0.6) | 15  (1.9) | 8  (0.7) | 9  (0.8) | 32  (1.9) | 24  (1.4) |
|  | Mental and behavioural disorders | 5  (0.4) | 4  (0.3) | 5  (0.5) | 5  (0.5) | 7  (0.5) | 4  (0.3) | 5  (0.6) | 4  (0.5) | 2  (0.2) | 5  (0.4) | 15  (0.9) | 4  (0.2) |
|  | Diseases of the nervous system | 125  (8.8) | 128  (9.0) | 132  (12.0) | 115  (10.5) | 114  (9.0) | 103  (8.1) | 59  (7.4) | 69  (8.7) | 110  (9.7) | 109  (9.6) | 149  (8.7) | 134  (7.9) |
|  | Diseases of the eye, adnex, ear and mastoid process | 1  (0.1) | 1  (0.1) | 0  (0.00) | 5  (0.5) | 0  (0.0) | 3  (0.2) | 1  (0.1) | 0  (0.0) | 2  (0.2) | 1  (0.1) | 0  (0.0) | 4  (0.2) |
|  | Diseases of the circulation system | 50  (3.5) | 46  (3.2) | 71  (6.5) | 63  (5.7) | 51  (4.0) | 53  (4.2) | 61  (7.7) | 35  (4.4) | 42  (3.7) | 47  (4.1) | 92  (5.4) | 93  (5.5) |
|  | Diseases of the respiratory system | 168  (11.9) | 163  (11.5) | 225  (20.5) | 196  (17.9) | 115  (9.0) | 139  (10.9) | 202  (25.5) | 186  (23.5) | 136  (12.0) | 145  (12.8) | 215  (12.7) | 218  (12.8) |
|  | Diseases of the digestsive system | 66  (4.7) | 72  (5.1) | 44  (4.0) | 52  (4.7) | 118  (9.3) | 121  (9.5) | 24  (3.0) | 37  (4.7) | 46  (4.1) | 58  (5.1) | 120  (7.0) | 136  (8.0) |
|  | Diseases of the skin and suscutaneous tissue | 4  (0.3) | 4  (0.3) | 2  (0.2) | 3  (0.3) | 2  (0.2) | 4  (0.3) | 2  (0.2) | 3  (0.4) | 5  (0.4) | 0  (0.0) | 5  (0.3) | 4  (0.2) |
|  | Diseases of the musculoskeletal system and connective tissue | 10  (0.7) | 5  (0.4) | 8  (0.7) | 7  (0.6) | 9  (0.7) | 11  (0.9) | 8  (1.0) | 5  (0.6) | 7  (0.6) | 4  (0.4) | 22  (1.3) | 9  (0.5) |
|  | Diseases of the genitourinary system | 58  (4.1) | 75  (5.3) | 9  (0.8) | 18  (1.6) | 24  (1.9) | 44  (3.5) | 10  (1.3) | 17  (2.1) | 54  (4.8) | 68  (6.0) | 92  (5.4) | 88  (5.2) |
|  | Certain conditions originating in the perinatal period | 13  (0.9) | 10  (0.7) | 9  (0.8) | 8  (0.7) | 10  (0.8) | 5  (0.4) | 2  (0.3) | 4  (0.5) | 2  (0.2) | 9  (0.8) | 17  (1.0) | 28  (1.6) |
|  | Congenital malformations, deformations and chromosomal abnormalities | 457  (32.3) | 465  (32.8) | 281  (25.6) | 299  (27.3) | 456  (35.8) | 450  (35.3) | 190  (24.0) | 185  (23.4) | 352  (31.0) | 351  (31.0) | 500  (29.3) | 508  (29.8) |
|  | Symptoms, signs and abnormal clinical and laboratory findings, not elsewhere classified | 86  (6.1) | 82  (5.8) | 50  (4.6) | 50  (4.6) | 62  (4.9) | 60  (4.7) | 38  (4.8) | 39  (4.9) | 78  (6.9) | 71  (6.3) | 88  (5.2) | 82  (4.8) |
|  | injury,poisoning and certain other consequrences of external causes | 123  (8.7) | 123  (8.7) | 85  (7.8) | 66  (6.0) | 68  (5.3) | 68  (5.3) | 61  (7.7) | 55  (6.9) | 79  (7.0) | 57  (5.0) | 116  (6.8) | 109  (6.4) |
|  | External causes of morbididty and mortality | 6  (0.4) | 1  (0.1) | 4  (0.4) | 1  (0.1) | 7  (0.5) | 1  (0.1) | 5  (0.6) | 3  (0.4) | 6  (0.5) | 2  (0.2) | 6  (0.4) | 4  (0.2) |
|  | Factors influencing health status and contact with health services | 3  (0.2) | 8  (0.6) | 2  (0.2) | 3  (0.3) | 5  (0.4) | 2  (0.2) | 2  (0.3) | 3  (0.4) | 4  (0.4) | 0  (0.0) | 5  (0.3) | 3  (0.2) |
|  | *P* | 0.754 | | 0.186 | | 0.213 | | 0.173 | | 0.102 | | 0.071 | |
| Length of hospital (mean (SD)) |  | 11.85  (8.83) | 11.67  (8.03) | 12.46  (9.43) | 12.00  (9.02) | 13.29  (14.33) | 13.20  (12.50) | 14.49  (11.84) | 13.55  (10.62) | 13.08  (8.60) | 12.64  (8.54) | 12.32  (9.77) | 11.96  (9.37) |
|  | *P* | 0.570 | | 0.241 | | 0.864 | | 0.097 | | 0.219 | | 0.277 | |

**Table S4 The clinical characteristic of patients in exposed group and unexposed group (Continued)**

| Factors |  | Methylprednisolone Sodium Succinate for Injection | | Compound Glycyrrhizin Injection | | Vancomycin Hydrochloride for Intra Venous | | Adrenaline Hydrochlaride Injection | | Milrinone Lactate Injection | | Meropenem for Injection | |
| --- | --- | --- | --- | --- | --- | --- | --- | --- | --- | --- | --- | --- | --- |
|  |  | EG | UEG | EG | UEG | EG | UEG | EG | UEG | EG | UEG | EG | UEG |
| n |  | 1162 | 1162 | 738 | 738 | 468 | 468 | 1222 | 1222 | 506 | 506 | 803 | 803 |
| caliper |  | 0.00003 | | 0.0010 | | 0.0004 | | 0.0004 | | 0.0011 | | 0.0006 | |
| Age (%) | 1 month - 1 year | 468 (40.3) | 469 (40.4) | 317 (43.0) | 334  (45.3) | 270 (57.7) | 278 (59.4) | 682 (55.8) | 687 (56.2) | 392 (77.5) | 390 (77.1) | 464 (57.8) | 476 (59.3) |
|  | 1 year – 10 years | 431 (37.1) | 429 (36.9) | 256 (34.7) | 242  (32.8) | 148 (31.6) | 146 (31.2) | 462 (37.8) | 457 (37.4) | 100 (19.8) | 104 (20.6) | 293 (36.5) | 279 (34.7) |
|  | 11 years – 18 years | 263 (22.6) | 264 (22.7) | 165 (22.4) | 162  (22.0) | 50 (10.7) | 44 (9.4) | 78 (6.4) | 78 (6.4) | 14 (2.8) | 12 (2.4) | 46 (5.7) | 48 (6.0) |
|  | *P* | 0.996 | | 0.649 | | 0.774 | | 0.978 | | 0.888 | | 0.764 | |
| Gender (%) | Male | 670 (57.7) | 670 (57.7) | 440 (59.6) | 415 (56.2) | 271 (57.9) | 294 (62.8) | 748 (61.2) | 729 (59.7) | 260 (51.4) | 274 (54.2) | 471 (58.7) | 483 (60.1) |
|  | Female | 556 (43.4) | 558 (43.6) |  |  |  |  |  |  |  |  |  |  |
|  | *P* | 1.000 | | 0.206 | | 0.142 | | 0.457 | | 0.413 | | 0.576 | |
| Major dignosis  (%) | Certain infectious and parastic diseases | 47 (4.0) | 47 (4.0) | 44 (6.0) | 40 (5.4) | 46 (9.8) | 48 (10.3) | 48 (3.9) | 52 (4.3) | 13 (2.6) | 10 (2.0) | 56 (7.0) | 59 (7.3) |
|  | Neoplasms | 90 (7.7) | 90 (7.7) | 68 (9.2) | 78 (10.6) | 67 (14.3) | 68 (14.5) | 111 (9.1) | 106 (8.7) | 9 (1.8) | 12 (2.4) | 87 (10.8) | 83 (10.3) |
|  | Diseases of the blood and blood-forming organs involving the immune mechanism | 20 (1.7) | 20 (1.7) | 34 (4.6) | 28 (3.8) | 14 (3.0) | 15 (3.2) | 30 (2.5) | 28 (2.3) | 2 (0.4) | 2 (0.4) | 31 (3.9) | 30 (3.7) |
|  | Endocrine,nutritional and metabolic diseases | 1 (0.1) | 1 (0.1) | 12 (1.6) | 9 (1.2) | 6 (1.3) | 13 (2.8) | 9 (0.7) | 11 (0.9) | 2 (0.4) | 3 (0.6) | 10 (1.2) | 22 (2.7) |
|  | Mental and behavioural disorders | 2 (0.2) | 2 (0.2) | 4 (0.5) | 1  (0.1) | 1 (0.2) | 0 (0.0) | 2 (0.2) | 4 (0.3) | 0 (0.0) | 0 (0.0) | 2 (0.2) | 1 (0.1) |
|  | Diseases of the nervous system | 101 (8.7) | 101 (8.7) | 57 (7.7) | 64 (8.7) | 42 (9.0) | 42 (9.0) | 144 (11.8) | 134 (11.0) | 5 (1.0) | 6 (1.2) | 86 (10.7) | 82 (10.2) |
|  | Diseases of the eye, adnex, ear and mastoid process | 0 (0.0) | 0 (0.0) | 0 (0.0) | 0  (0.0) | 0 (0.0) | 0 (0.0) | 2 (0.2) | 1 (0.1) | 0 (0.0) | 0 (0.0) | 0 (0.0) | 0 (0.0) |
|  | Diseases of the circulation system | 49 (4.2) | 49 (4.2) | 34 (4.6) | 39 (5.3) | 12 (2.6) | 13 (2.8) | 78 (6.4) | 59 (4.8) | 48 (9.5) | 26 (5.1) | 31 (3.9) | 29 (3.6) |
|  | Diseases of the respiratory system | 187 (16.1) | 187 (16.1) | 91 (12.3) | 120 (16.3) | 112 (23.9) | 116 (24.8) | 208 (17.0) | 209 (17.1) | 63 (12.5) | 77 (15.2) | 160 (19.9) | 163 (20.3) |
|  | Diseases of the digestsive system | 32 (2.8) | 32 (2.8) | 103 (14.0) | 82 (11.1) | 24 (5.1) | 26 (5.6) | 100 (8.2) | 117 (9.6) | 2 (0.4) | 4 (0.8) | 75 (9.3) | 79 (9.8) |
|  | Diseases of the skin and suscutaneous tissue | 2 (0.2) | 2 (0.2) | 3 (0.4) | 1 (0.1) | 1 (0.2) | 3 (0.6) | 3 (0.2) | 6 (0.5) | 0 (0.0) | 1 (0.2) | 6 (0.7) | 0 (0.0) |
|  | Diseases of the musculoskeletal system and connective tissue | 0 (0.0) | 0 (0.0) | 6 (0.8) | 6 (0.8) | 7 (1.5) | 0 (0.0) | 6 (0.5) | 6 (0.5) | 1 (0.2) | 2 (0.4) | 7 (0.9) | 3 (0.4) |
|  | Diseases of the genitourinary system | 14 (1.2) | 14 (1.2) | 10 (1.4) | 19 (2.6) | 6 (1.3) | 8 (1.7) | 21 (1.7) | 51 (4.2) | 2 (0.4) | 2 (0.4) | 47 (5.9) | 41 (5.1) |
|  | Certain conditions originating in the perinatal period | 3 (0.3) | 3 (0.3) | 14 (1.9) | 8 (1.1) | 6 (1.3) | 4 (0.9) | 9 (0.7) | 11 (0.9) | 1 (0.2) | 7 (1.4) | 5 (0.6) | 6 (0.7) |
|  | Congenital malformations, deformations and chromosomal abnormalities | 464 (39.9) | 463 (39.8) | 106 (14.4) | 122 (16.5) | 53 (11.3) | 53 (11.3) | 245 (20.0) | 241 (19.7) | 350 (69.2) | 337 (66.6) | 92 (11.5) | 110 (13.7) |
|  | Symptoms, signs and abnormal clinical and laboratory findings, not elsewhere classified | 60 (5.2) | 61 (5.2) | 58 (7.9) | 51 (6.9) | 25 (5.3) | 24 (5.1) | 79 (6.5) | 78 (6.4) | 6 (1.2) | 11 (2.2) | 46 (5.7) | 42 (5.2) |
|  | injury,poisoning and certain other consequrences of external causes | 84 (7.2) | 85 (7.2) | 84 (11.4) | 63 (8.5) | 40 (8.5) | 33 (7.1) | 119 (9.7) | 105 (8.6) | 2 (0.4) | 2 (0.4) | 58 (7.2) | 49 (6.1) |
|  | External causes of morbididty and mortality | 6 (0.5) | 5 (0.4) | 6 (0.8) | 6 (0.8) | 3 (0.6) | 2 (0.4) | 4 (0.3) | 2 (0.2) | 0 (0.0) | 3 (0.6) | 3 (0.4) | 0 (0.0) |
|  | Factors influencing health status and contact with health services | 0 (0.0) | 0 (0.0) | 4 (0.5) | 1 (0.1) | 3 (0.6) | 0 (0.0) | 4 (0.3) | 1 (0.1) | 0 (0.0) | 1 (0.2) | 1 (0.1) | 4 (0.5) |
|  | *P* | 1.000 | | 0.029 | | 0.494 | | 0.192 | | 0.168 | | 0.237 | |
| Length of hospital (mean (SD)) |  | 10.90 (7.63) | 10.90 (7.63) | 13.85 (9.67) | 13.30 (9.47) | 14.88 (11.27) | 14.03 (11.22) | 12.06 (10.41) | 11.76 (9.91) | 15.54 (9.43) | 15.40 (9.94) | 13.09 (10.09) | 12.78 (10.11) |
|  | *P* | 1.000 | | 0.270 | | 0.248 | | 0.457 | | 0.825 | | 0.535 | |

**Table S4 The clinical characteristic of patients in exposed group and unexposed group (Continued)**

| Factors |  | Human Immumoglobulin for Intravenous Injection | | Aminomethylbenzoic Acid Injection | | Etamsylate Injection | |
| --- | --- | --- | --- | --- | --- | --- | --- |
|  |  | EG | UEG | EG | UEG | EG | UEG |
| n |  | 688 | 688 | 804 | 804 | 1015 | 1015 |
| caliper |  | 0.0003 | | 0.0007 | | 0.0012 | |
| Age | 1 month - 1 year | 433 (62.9) | 448 (65.1) | 261 (32.5) | 267 (33.2) | 361 (35.6) | 387 (38.1) |
|  | 1 year – 10 years | 223 (32.4) | 209 (30.4) | 370 (46.0) | 367 (45.6) | 431 (42.5) | 405 (39.9) |
|  | 11 years – 18 years | 32 (4.7) | 31 (4.5) | 173 (21.5) | 170 (21.1) | 223 (22.0) | 223 (22.0) |
|  | *P* | 0.696 | | 0.948 | | 0.425 | |
| Gender (%) | Male | 405 (58.9) | 386 (56.1) | 492 (61.2) | 501 (62.3) | 610 (60.1) | 624 (61.5) |
|  | Female |  |  |  |  |  |  |
|  | *P* | 0.326 | | 0.681 | | 0.555 | |
| Major dignosis  (%) | Certain infectious and parastic diseases | 44 (6.4) | 48 (7.0) | 28 (3.5) | 50 (6.2) | 32 (3.2) | 38 (3.7) |
|  | Neoplasms | 37 (5.4) | 38 (5.5) | 126 (15.7) | 126 (15.7) | 120 (11.8) | 112 (11.0) |
|  | Diseases of the blood and blood-forming organs involving the immune mechanism | 38 (5.5) | 35 (5.1) | 72 (9.0) | 45 (5.6) | 50 (4.9) | 37 (3.6) |
|  | Endocrine,nutritional and metabolic diseases | 2 (0.3) | 14 (2.0) | 9 (1.1) | 7 (0.9) | 11 (1.1) | 16 (1.6) |
|  | Mental and behavioural disorders | 1 (0.1) | 2 (0.3) | 3 (0.4) | 4 (0.5) | 2 (0.2) | 10 (1.0) |
|  | Diseases of the nervous system | 108 (15.7) | 101 (14.7) | 52 (6.5) | 58 (7.2) | 60 (5.9) | 70 (6.9) |
|  | Diseases of the eye, adnex, ear and mastoid process | 0 (0.0) | 1 (0.1) | 0 (0.0) | 0 (0.0) | 2 (0.2) | 4 (0.4) |
|  | Diseases of the circulation system | 36 (5.2) | 29 (4.2) | 57 (7.1) | 57 (7.1) | 56 (5.5) | 67 (6.6) |
|  | Diseases of the respiratory system | 178 (25.9) | 174 (25.3) | 46 (5.7) | 48 (6.0) | 61 (6.0) | 77 (7.6) |
|  | Diseases of the digestsive system | 29 (4.2) | 35 (5.1) | 91 (11.3) | 91 (11.3) | 118 (11.6) | 92 (9.1) |
|  | Diseases of the skin and suscutaneous tissue | 3 (0.4) | 2 (0.3) | 4 (0.5) | 0 (0.0) | 1 (0.1) | 2 (0.2) |
|  | Diseases of the musculoskeletal system and connective tissue | 8 (1.2) | 3 (0.4) | 9 (1.1)" | 5 (0.6) | 6 (0.6) | 3 (0.3) |
|  | Diseases of the genitourinary system | 6 (0.9) | 10 (1.5) | 86 (10.7) | 77 (9.6) | 128 (12.6) | 108 (10.6) |
|  | Certain conditions originating in the perinatal period | 1 (0.1) | 2 (0.3) | 3 (0.4) | 6 (0.7) | 4 (0.4) | 6 (0.6) |
|  | Congenital malformations, deformations and chromosomal abnormalities | 154 (22.4) | 155 (22.5) | 97 (12.1) | 117 (14.6) | 176 (17.3) | 205 (20.2) |
|  | Symptoms, signs and abnormal clinical and laboratory findings, not elsewhere classified | 27 (3.9) | 27 (3.9) | 51 (6.3) | 47 (5.8) | 75 (7.4) | 67 (6.6) |
|  | injury,poisoning and certain other consequrences of external causes | 12 (1.7) | 11 (1.6) | 60 (7.5) | 60 (7.5) | 97 (9.6) | 92 (9.1) |
|  | External causes of morbididty and mortality | 2 (0.3) | 1 (0.1) | 8 (1.0) | 4 (0.5) | 11 (1.1) | 6 (0.6) |
|  | Factors influencing health status and contact with health services | 2 (0.3) | 0 (0.0) | 2 (0.2) | 2 (0.2) | 5 (0.5) | 3 (0.3) |
|  | *P* | 0.428 | | 0.143 | | 0.134 | |
| Length of hospital (mean (SD)) |  | 14.24 (9.49) | 13.77 (9.26) | 13.36 (11.82) | 13.13 (13.68) | 13.00 (10.59) | 12.51 (11.37) |
|  | *P* | 0.352 | | 0.721 | | 0.312 | |

**Table S5 Mortality between exposed and unexposed of suspected drugs groups**

| Drug ID | Drugs name | Exposed group  n (%) | Unexposed group  n (%) | P |
| --- | --- | --- | --- | --- |
| 1 | Furosemide Injection | 178 (12.6) | 47 (3.3) | <0.001 |
| 2 | Midazolam Injection | 229 (20.9) | 60 (5.5) | <0.001 |
| 3 | 20% Albumin Prepared From Human Plasma Injection | 180 (14.1) | 72 (5.7) | <0.001 |
| 4 | Fentanyl Citrate Injection | 214 (27.0) | 48 (6.1) | <0.001 |
| 5 | Ibuprofen Suspension | 114 (10.1) | 66 (5.8) | <0.001 |
| 6 | Diazepam Injection | 173 (10.2) | 134 (7.9) | 0.020 |
| 7 | Methylprednisolone Sodium Succinate for Injection | 156 (13.4) | 58 (5.0) | <0.001 |
| 8 | Compound Glycyrrhizin Injection | 106 (14.4) | 72 (9.8) | 0.007 |
| 9 | Vancomycin Hydrochloride for Intra Venous | 124 (26.5) | 50 (10.7) | <0.001 |
| 10 | Adrenaline Hydrochlaride Injection | 210 (17.2) | 65 (5.3) | <0.001 |
| 11 | Milrinone Lactate Injection | 44 (8.7) | 40 (7.9) | 0.649 |
| 12 | Meropenem for Injection | 167 (20.8) | 71 (8.8) | <0.001 |
| 13 | Human Immumoglobulin for Intravenous Injection | 126 (18.3) | 64 (9.3) | <0.001 |
| 14 | Aminomethylbenzoic Acid Injection | 130 (16.2) | 60 (7.5) | <0.001 |
| 15 | Etamsylate Injection | 116 (11.4) | 78 (7.7) | 0.004 |
